# Universal CAR T cells targeted to HER2 with a biotin-trastuzumab soluble linker penetrate spheroids and large tumor xenografts that are inherently resistant to trastuzumab mediated ADCC

**DOI:** 10.3389/fimmu.2024.1365172

**Published:** 2024-03-18

**Authors:** Lőrinc Nagy, Marianna Mezősi-Csaplár, István Rebenku, György Vereb, Árpád Szöőr

**Affiliations:** ^1^ Department of Biophysics and Cell Biology, Faculty of Medicine, University of Debrecen, Debrecen, Hungary; ^2^ HUN-REN-UD Cell Biology and Signaling Research Group, Faculty of Medicine, University of Debrecen, Debrecen, Hungary; ^3^ Faculty of Pharmacy, University of Debrecen, Debrecen, Hungary

**Keywords:** breast cancer, HER2, trastuzumab, universal chimeric antigen receptor, immunotherapy, cell therapy

## Abstract

CAR T cell therapies face challenges in combating solid tumors due to their single-target approach, which becomes ineffective if the targeted antigen is absent or lost. Universal CAR T cells (UniCAR Ts) provide a promising solution by utilizing molecular tags (linkers), such as biotin conjugated to monoclonal antibodies, enabling them to target a variety of tumor antigens. Recently, we showed that conventional CAR T cells could penetrate the extracellular matrix (ECM) of ADCC-resistant tumors, which forms a barrier to therapeutic antibodies. This finding led us to investigate whether UniCAR T cells, targeted by soluble antibody-derived linkers, could similarly tackle ADCC-resistant tumors where ECM restricts antibody penetration. We engineered UniCAR T cells by incorporating a biotin-binding monomeric streptavidin 2 (mSA2) domain for targeting HER2 via biotinylated trastuzumab (BT). The activation and cytotoxicity of UniCAR T cells in the presence or absence of BT were evaluated in conventional immunoassays. A 3D spheroid coculture was set up to test the capability of UniCAR Ts to access ECM-masked HER2^+^ cells. For *in vivo* analysis, we utilized a HER2^+^ xenograft model in which intravenously administered UniCAR T cells were supplemented with intraperitoneal BT treatments. *In vitro*, BT-guided UniCAR T cells showed effective activation and distinct anti-tumor response. Upon target recognition, IFNγ secretion correlated with BT concentration. In the presence of BT, UniCAR T cells effectively penetrated HER2^+^ spheroids and induced cell death in their core regions. *In vivo*, upon intravenous administration of UniCAR Ts, circulating BT linkers immediately engaged the mSA2 domain and directed effector cells to the HER2^+^ tumors. However, these co-treated mice died early, possibly due to the lung infiltration of UniCAR T cells that could recognize both native biotin and HER2. Our results suggest that UniCAR T cells guided with soluble linkers present a viable alternative to conventional CAR T cells, especially for patients resistant to antibody therapy and those with solid tumors exhibiting high antigenic variability. Critical to their success, however, is the choice of an appropriate binding domain for the CAR and the corresponding soluble linker, ensuring both efficacy and safety in therapeutic applications.

## 1 Introduction

In the last decade, the development of chimeric antigen receptor (CAR) T cell products has represented a paradigm shift in the treatment of chemotherapy-resistant leukemias and lymphomas (1, 2), however, clinical efficacy in solid tumor trials has been sobering. CARs bind to the target antigen and activate the immune cells expressing them, leading to consequential cytokine production, lysis of target cells, and expansion. An important point of concern is the single specificity of CAR T cells that can make therapy ineffective due to antigen loss or tumor heterogeneity (3). In addition, in many patients participating in clinical trials, the unpredictable and uncontrollable activation and expansion of CAR T cells have led to the development of severe side effects, primarily based on excessive cytokine release syndrome (CRS) (4). In contrast to conventional CAR T cells, universal CAR T cells (UniCAR Ts) that bind to the target antigen by recognizing a molecular tag (e.g. FITC, peptide neo-epitopes or biotin) - which is conjugated to a monoclonal antibody to constitute a soluble linker (also termed a molecular switch, or linker)- might overcome these limitations (5–8). In these approaches, sequential or simultaneous co-administration of various tagged antibodies offers the prospect of parallelly targeting multiple tumor antigens. In addition, the activity of UniCAR T cells can be fine-tuned by modulating the concentration of the linker molecules. Furthermore, some of the side effects can be eliminated by suspending their administration. UniCAR T cells are actively tested in two ongoing clinical trials against AML (NCT04450069) and renal or prostate cancer (NCT04633148).

Recently, we confirmed that a single dose of trastuzumab-derived HER2-specific CAR T cells, as actively moving living drugs, could penetrate and eradicate established trastuzumab-resistant MDA-HER2 and JIMT-1 tumor xenografts while saturating doses of soluble trastuzumab co-administered with multiple doses of CD16.176V.NK-92 effector cells only transiently retarded tumor growth (9). Our data suggest that conventional CAR T cells expressing membrane-bound TAA-specific scFvs as extracellular recognition domains can penetrate solid tumors with an extensive extracellular matrix that masks the target antigen (10), thereby successfully eliminating antibody therapy-resistant tumors (9). This finding raises the question whether UniCAR T cells, targeted by soluble antibody-derived linker molecules, can induce anti-tumor effects in therapy-resistant tumors in which a massive extracellular matrix has been established that restricts antibody binding.

To address this question, we have engineered T cells expressing UniCARs that use an affinity-enhanced monomeric streptavidin 2 (mSA2) biotin-binding domain (7) as an extracellular recognition unit that could target HER2^+^ tumor cells through biotinylated trastuzumab (BT). In the presence of BT, these UniCAR T cells recognize and kill three-dimensional cultures of MDA-HER2 breast cancer cells, which are inherently resistant to trastuzumab (9). *In vivo*, we could confirm that upon intravenous administration of UniCAR T cells, circulating soluble BT linkers immediately engaged the mSA2 domain and directed UniCAR T cells towards MDA-HER2 xenografts, which they penetrated. However, our data reveal that co-administration of streptavidin-derived UniCARs with biotinylated trastuzumab poses a safety risk due to the recognition of biotin-accumulating and/or HER2-expressing cells in the lungs.

## 2 Methods

All materials were from Sigma-Aldrich (St. Louis, MO, USA), unless otherwise indicated.

### 2.1 Cells and culture condition

HEK 293T packaging cells were purchased from the American Type Culture Collection (ATCC, Manassas, VA, USA); the triple-negative human breast cancer cell line MDA-MB-468 (abbreviated MDA) and its variant stably expressing HER2 (MDA-HER2) (11) were a kind gift of Stephen Gottschalk (St. Jude Children’s Hospital; Memphis; TN; USA). Cells were cultured in Dulbecco’s Modified Eagle Medium (DMEM) supplemented with 2 mmol/l GlutaMAX, 10% Fetal Calf Serum (FCS) and antibiotics. Primary human T cells, UniCAR and HER2-specific CAR T cells were cultured in RPMI (Roswell Park Memorial Institute) medium supplemented with 2 mmol/l GlutaMAX, 10% FCS and antibiotics.

All cells and cell lines listed above were maintained in a humidified atmosphere containing 5% CO_2_ at 37°C and were routinely checked for the absence of Mycoplasma contamination. MDA.ffLUC and MDA-HER2.ffLUC cell lines were generated by single-cell cloning of MDA-MB-468 and MDA-HER2 cell lines, respectively, after transduction with a retrovirus encoding eGFP.ffLUC to express an enhanced green fluorescent protein/firefly luciferase fusion gene (9).

### 2.2 Retrovirus production and transduction of T cells

Retroviral particles were generated by transient transfection of HEK 293T cells with CAR coding retroviral transfer vectors, the Peg-Pam-e plasmid containing the sequence for MoMLV gag-pol, and the pMax.RD114 plasmid containing the sequence for RD114 by using jetPrime transfection reagent (Poly-plus, Illkirch, France). The helper plasmids were kind gifts of Stephen Gottschalk (St.Jude Children’s Hospital; Memphis; TN; USA). To generate a pSFG retroviral UniCAR construct, we incorporated the mSA2-derived recognition domain (kindly provided by Jason J. Lohmueller (7)) into the CAR backbone consisting of the IgG heavy chain signal peptide; the IgG1 short hinge, a transmembrane region of human CD28 with CD28 intracellular costimulatory endo domain and the cytoplasmic region of human CD3ζ (12). The trastuzumab-derived HER2-specific CAR, a kind gift from Dr. Richard A. Morgan at the NCI, NIH, is coded in an MSGV vector and is composed of the Igκ chain signal peptide; trastuzumab light chain variable region; the 218 peptide linker; trastuzumab heavy chain variable region; the hinge, transmembrane and cytoplasmic regions of human CD28; and the cytoplasmic region of human CD3ζ (13, 14). Supernatants containing the retrovirus particles were collected after 48 hours.

Experiments were carried out on human samples in accordance with the Declaration of Helsinki and approved by the Regional and Institutional Committee for Research Ethics (RKEB.5378/2019). To generate CAR T cells, human peripheral blood mononuclear cells were isolated by Ficoll gradient centrifugation and stimulated in non-tissue culture 24-well plates pre-coated with 1 µg/ml OKT3 (Thermo Fischer, Waltham, MA, USA) and anti-CD28 (R&D Systems, Minneapolis, MN, USA) antibodies. On day 2, human interleukin-7 (IL-7; 10 ng/ml) and human interleukin-15 (IL-15; 5 ng/ml) (Miltenyi Biotec, Bergisch Gladbach, Germany) were added to cultures. T cells were transduced with retroviral particles on RetroNectin-coated (Takara, Kusatsu, Japan) plates on day 3 in the presence of IL-7 (10 ng/ml) and IL-15 (5 ng/ml). The expansion of T cells was subsequently supported with IL-7 and IL-15. OKT3/CD28 activated non-transduced (NT) T cells were expanded with IL-7 and IL-15 using the same protocol. Following 48h incubation, cells were used for further experiments.

### 2.3 Flow cytometry

In cell products, HER2-specific CAR expression was confirmed by labeling with a HER2-Fc fusion protein (R&D Systems, Minneapolis, MN, USA) followed by Alexa Fluor 647 conjugated anti-human IgG (Invitrogen/Thermo Fisher/, Carlsbad, CA, USA), UniCAR expression was confirmed by labeling with biotinylated highly cross-absorbed goat-anti-chicken IgG conjugated with Alexa Fluor 647. T cell purity was determined by Alexa Fluor 488 conjugated anti-human CD3 antibody (BD Biosciences, San Jose, CA, USA) staining.

In murine blood samples, human T cells were identified based on their pre-loaded CFSE signal and by labeling with APC-conjugated anti-human CD45 antibody (BD Biosciences, San Jose, CA, USA) antibody. The presence of conventional HER2-CARs or biotinylated trastuzumab-bound UniCAR molecules in the membrane of human T lymphocytes was confirmed by labeling with an Alexa Fluor 647 conjugated monomeric HER2 extracellular domain (Sino Biological Europe GmbH, Eschborn, Germany) in order to achieve comparable signals for the two cell types in a single labeling step.

Conjugation of the biotin and HER2 with Alexa Fluor 647 (Thermo Fisher Scientific, Waltham, MA, USA) was carried out according to the manufacturers’ specifications and as previously described (15). For the biotinylation of trastuzumab, a standard protein modification protocol was used. The antibody was dissolved in PBS (pH 7.0) at a concentration of 1 mg/ml. The molar ratio in the reaction mixture was 5:1 of biotinyliating reagent (NHS-Biotin 1 mg/ml in DMSO) to trastuzumab. The reaction took place under constant stirring at room temperature for 30 min. Biotinylated trastuzumab was purified using protein A affinity chromatography.

All molecules were used at 10 µg/ml final concentration for 10 minutes on ice. Analysis was performed on at least 10,000 cells per sample using a NovoCyte 3000RYB (ACEA Biosciences, San Diego, CA, USA) instrument and NovoExpress software (ACEA Biosciences, San Diego, CA, USA).

### 2.4 Western blot

Expression of CARs was confirmed by western blot, using an anti-human CD3ζ antibody for detection. t. HER2-CAR, UniCAR and NT T cells were washed with PBS and whole cell lysates were prepared in a lysis buffer containing 50 mM Tris-HCl, 150 mM NaCl, 0.1% Triton X-100, 5 mM EDTA and a protease inhibitor cocktail of 2 mM PMSF, 1 mM sodium orthovanadate, and 1X cOmplete™ Mini Protease Inhibitor Cocktail tablet (Roche, Basel, Switzerland). Cell lysates were resolved on SDS-polyacrylamide gels in a reducing loading buffer containing 0.1 mM DTT and blotted to PVDF membranes. Membranes were blocked with 5% milk in Tris-buffered saline containing 0.1% Tween20 for 1 hour at room temperature and washed in PBS. The membranes were then probed with 1 µg/ml mouse anti-human CD3ζ (BD Biosciences, San Jose, CA, USA) antibody overnight at 4°C. Membranes were washed and probed with a secondary anti-mouse IgG–horseradish peroxidase (HRP) for 1 hour at room temperature.

### 2.5 Cytokine secretion assay

HER2-specific CAR T cells or UniCAR T cells (in the presence or absence of various concentrations of biotinylated trastuzumab) were plated onto 1 µg/ml HER2-Fc (R&D Systems, Minneapolis, MN, USA) pre-coated plates or cocultured with MDA-HER2 target cells (in the presence or absence of 10 µg/ml biotinylated trastuzumab) at a 1:1 effector to target ratio. Following 24 hours of culture, the supernatant was harvested and analyzed for the presence of interferon-gamma (IFNγ) by ELISA (R&D systems, Minneapolis, MN, USA) according to the manufacturer’s instruction using a Spark® multimode microplate reader (Tecan Group Ltd., Männedorf, Switzerland). The HER2^-^ MDA-MB-468 cell line and NT T cells served as controls.

### 2.6 Cytotoxicity assay

Cytotoxic activity of various CAR T cells against targets was determined by a luciferase-based cytotoxicity assay (9). MDA and MDA-HER2 cells expressing eGFP/ffLUC were plated in 96-well flat bottom plates at a concentration of 3 × 10^4^ cells/well in quadruplets. After 24h, various effector cells were added at a 1:1 effector to tumor cell ratio in the presence or absence of 10 µg/ml of biotinylated trastuzumab. Wells without effector cells served as untreated control references. After 24h, luciferase activity was determined using a luciferase assay kit according to the manufacturer’s instructions (Promega, Madison, WI, USA) and a Synergy HT luminometer (BioTek, Winooski, VE, USA).

### 2.7 *In vitro* rechallenge assay

HER2-specific CAR T cells or UniCAR T cells (in the presence or absence of 10 µg/ml biotinylated trastuzumab; 2 × 10^5^ cells/well) were placed onto flat-bottom plates pre-coated with 1 µg/ml HER2-Fc (R&D Systems, Minneapolis, MN, USA). Every 3.5 days, the effector cell number was determined by flow cytometry, and then the proliferation rate was calculated by dividing the total effector cell number on the present day by the number of effector cells plated at the beginning of the last 3.5-day round. A new round was initiated by re-plating 2 × 10^5^ effector cells onto freshly coated plates. The effector cells did not receive interleukin supplements during the experiment. If the proliferation rate fell below 1, then the available maximum amount of effector cells was placed onto fresh plates at the beginning of a new round. The experiment was concluded for any subset when the proliferation rate of the effector cells fell under 0.45.

### 2.8 Three-dimensional cell culture and propidium iodide incorporation assay

MDA-HER2.ffLUC cells (1 × 10^5^ cells/ml in 200 μl) were placed into 96-well U-bottom plates in a cold medium containing 2.5% Matrigel (BD Biosciences, San Jose, CA, USA). The suspensions were centrifuged at 1000×*g* for 10 minutes at 10°C, and the cell pellet was cultured for 10 days. Cytotoxic activity of UniCAR T cells in these three-dimensional cell cultures was determined by propidium iodide incorporation assay (16). Spheroids of equal size were cocultured with 2 × 10^5^ effector cells. After 24 h, 3D cocultures were labeled with 1 µg/ml propidium iodide and target cell killing was measured with a Zeiss LSM 880 confocal microscope. A quantitative digital image processing pipeline (16), created in ImageJ (17), was used to calculate PI incorporation of MDA-HER2 cells in the entire spheroid and the inner core, which was defined to be within half the radius of the spheroid. PI incorporation was determined in these regions as integrated PI fluorescence intensity above the threshold in all background-corrected images. Two spheroids and at least five 2-µm-thick optical slices per sample were analyzed.

### 2.9 *In vivo* biotin binding experiment

NSG mice received a single i.v. dose of 20 × 10^6^ NT T cells (NT group) or HER2-CAR T cells (HER2-CAR group), or UniCAR T cells (UniCAR group). Mice co-treated with UniCAR T cells and biotinylated trastuzumab were treated with 100 µg biotinylated trastuzumab in 100 µl PBS i.p. 15 hours prior to injecting a single i.v. dose of 20 × 10^6^ UniCAR T cells. All administered human T cell products were pre-labeled with 1µM CFSE for 10 minutes on ice.

Retroorbital blood samples were taken from each mouse 2 minutes after T cell injection, and the animals were sacrificed.

The blood samples were mixed with RBC lysis puffer, washed with PBS twice, separated into two equal samples, and stained for human CD45 and for CAR T cells. Samples were stained and analyzed by flow cytometry as described above.

At termination, mice were dissected, lung samples were embedded in cryomatrix (Thermo Fischer Scientific, Waltham, MA, USA), and snap-frozen in isopentane cooled with liquid nitrogen.

### 2.10 Xenograft tumors and *in vivo* treatment

NSG (NOD.Cg-Prkdcscid/Il2rgtm1Wjl/SzJ) mice were purchased from The Jackson Laboratory and housed in a specific pathogen-free environment. All animal experiments were performed in accordance with FELASA guidelines and recommendations and DIN EN ISO 9001 standards. On day 0, each seven-week-old female NSG mouse participating in the study was given a subcutaneous injection in both flanks, each containing 3 × 10^6^ MDA-HER2.ffLUC cells in 100 µl PBS mixed with an equal volume of Matrigel (BD Biosciences, San Jose, CA, USA). Tumor growth was monitored with an IVIS Spectrum CT instrument (Perkin Elmer, Waltham, MA, USA). Before measurement, isoflurane-anesthetized animals were injected i.p. with D-luciferin (150 mg/kg). A bioluminescence image was obtained and analyzed after 10 minutes using Living Image software Version 4.0 (Caliper Life Sciences, Waltham, MA, USA). Signal intensity measured as total photons per second per square centimeter per steradian (p/(s x cm2 x sr)) was obtained from identically sized ROIs. Effector cell treated mice received on day 21 a single i.v. dose of 2 × 10^6^ NT T cells (NT group) or HER2-CAR T cells (HER2-CAR group), or UniCAR T cells (UniCAR group). Mice co-treated with UniCAR T cells and biotinylated trastuzumab received on day 21 a single i.v. dose of 2 × 10^6^ UniCAR T cells plus treated with 100 µg biotinylated trastuzumab in 100 µl PBS i.p twice weekly from this day (UniCAR+BT group). BT treated mice received 100 µg biotinylated trastuzumab in 100 µl PBS i.p. twice weekly from day 21 post tumor cell inoculation (BT group). (**Supplementary Figure 1**). Experiments were approved by the National Ethical Committee for Animal Research (# 5-1/2018/DEMÁB).

### 2.11 Tumor xenograft and tissue sections

At termination, mice were dissected, and fresh tumors and organs (heart, lung, liver, kidney, spleen) were embedded in cryomatrix (Thermo Fischer Scientific, Waltham, MA, USA) and snap-frozen in isopentane cooled with liquid nitrogen. Serial 4 µm thick cryosections were made with a Shandon Cryotome (Thermo Fischer Scientific, Waltham, MA, USA) at −24°C, and air-dried and stored at -20°C until further use. Cryosections were stained for human CD4^+^, CD8^+^ and CD45^+^ T cells.

All labeling molecules were diluted in PBS buffer supplemented with 1% BSA. After 5 min of rehydration of the cryosections in PBS buffer containing 1% BSA and 0.01% TritonX-100 (Thermo Fischer Scientific, Waltham, MA, USA), human T cells were stained with Alexa Fluor 647 conjugated rat anti-human CD8 (YTC 182.20; prepared from hybridoma supernatant; (18)), Alexa Fluor 488 conjugated rat anti-human CD4 (YNB 46.1.8; prepared from hybridoma supernatant; (18)) and APC conjugated anti-human CD45 (BioLegend, San Diego, California) antibodies. The possible presence of mouse T cells was investigated by staining with Alexa Fluor 647 conjugated rat anti-mouse CD8 (YTS 105.18.10; prepared from hybridoma supernatant) (19); Alexa Fluor 546 conjugated rat anti-mouse CD3 (145-2C11; prepared from CRL-1975 (ATCC) hybridoma supernatant) and Alexa Fluor 488 conjugated anti-mouse CD4 (YTS 177.96.1; prepared from hybridoma supernatant) (19). Mouse macrophages were stained with APC-conjugated F4/80 antibodies (BD Biosciences, San Jose, CA, USA), while mouse neutrophil granulocytes were stained with APC-conjugated Ly-6G rat antibodies (1A8-Ly6g, Thermo Fischer Scientific). HER2 expression was investigated with Alexa Fluor 647 conjugated anti-human HER2 mAb (ErbB2-76.5; produced from hybridoma supernatants (ErbB2-76.5, a kind gift from Y. Yarden, Weizmann Institute of Science, Rehovot, Israel)). The presence of biotin in organ samples was confirmed by FITC-conjugated extravidin. All antibodies were used at 10 µg/ml concentration on ice for 1 hour. Sections were washed for 5, 10, 5, and 10 minutes using DAPI at 1 µg/ml in the third wash buffer for nuclear staining and mounted in Mowiol antifade.

### 2.12 Hematoxylin-eosin staining

Samples were stained with the standard hematoxylin-eosin procedure and fixed with DPX. The prepared samples were scanned in with a Pannoramic confocal microscope at ×20 magnification. The composite images were handled by CaseViewer software (3DHISTECH, Budapest, Hungary).

### 2.13 Conventional light microscopy

UniCAR and HER2-CAR cocultures were analyzed with a Zeiss Axiovert 200M microscope (×10 objective). Images were taken with ZEN 2.6 Blue Edition software, postprocessing was handled with Fiji ImageJ 1.53t.

### 2.14 Confocal laser scanning microscopy

Fluorescence-labeled tissue sections were analyzed with a confocal laser scanning microscope (LSM 880, Carl Zeiss GmbH, Jena, Germany). Alexa Fluor 488 and EGFP were excited at 488 nm, Alexa Fluor 546 and propidium iodide (PI) at 543nm, APC and Alexa Fluor 647 at 633 nm. Corresponding fluorescence emission was separated with an appropriate quad-band dichroic mirror and detected on a 32-element GaAsP spectral detector in bands of 505 to 550 nm, 560 to 615 nm, and above 650 nm, respectively. For tissue samples, 5 consecutive, 2 µm thick optical sections were taken at 3 µm intervals, covering the central 10 µm part of the sections.

### 2.15 Statistical analysis

GraphPad Prism 5 software (GraphPad software, Inc., La Jolla, CA) was used for statistical analysis. Data were presented as mean ± SD or SEM. For comparison between two groups, a two-tailed t-test was used. One-way ANOVA with Bonferroni’s *post hoc* test was used to compare three or more groups. Survival, measured from the time of tumor cell injection, was analyzed by the Kaplan-Meier method and log-rank test. *P* values < 0.05 were considered statistically significant.

## 3 Results

As tumor models, we have used a HER2^+^ trastuzumab-resistant cell line, an *in vitro* generated MDA-MB-468 variant, MDA-HER2, stably expressing ectopic HER2 (9, 11). MDA-MB-468 (MDA for short) served as HER2^-^ control. Also, ffLUC-expressing variants of MDA and MDA-HER2 were used where appropriate.

### 3.1 Generation of UniCAR T cells expressing affinity-enhanced monomeric streptavidin 2 biotin-binding domain

**Figure 1:**
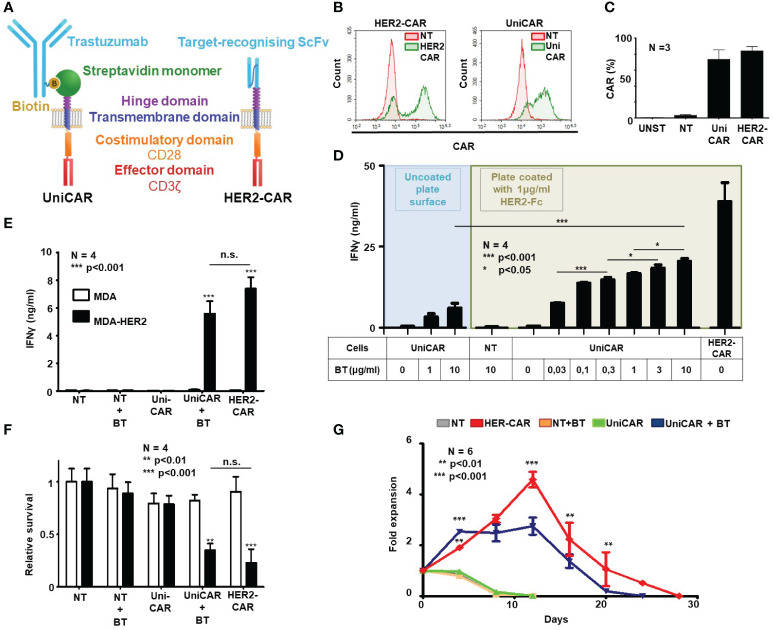
Generation of HER2-targeted human UniCAR and conventional CAR (HER2-CAR) T cells and characterization of their *in vitro* function. **(A)** Comparison of universal and conventional CARs recognizing HER2. UniCAR: the high-affinity monomeric streptavidin (mSA2) extracellular domain binds the HER2-recognizing biotinylated trastuzumab (BT) linker through a non-covalent interaction. Conventional HER2-CAR: it binds directly to HER2 by an extracellular domain derived from trastuzumab ScFv. Both constructs contain the same CD28 transmembrane and intracellular domains and the CD3ζ effector domain. **(B, C)** Representative flow cytometry histogram and summary data of CAR expression (N = 4 for each transduction; NT, non-transduced; UNST, unstained control). HER2-specific CAR expression was confirmed by labeling with a HER2-Fc fusion protein followed by Alexa Fluor 647 conjugated anti-human IgG, UniCAR expression was confirmed by labeling with biotinylated goat anti-chicken IgG conjugated with Alexa Fluor 647. **(D, E)** IFNγ-ELISA assays. T cell products were incubated with surface-adsorbed molecular HER2-Fc target and various BT concentrations **(D)** or with HER2+/- MDA target cells and a fixed BT concentration **(E)**. After 24h, IFNγ was determined in the culture supernatant by ELISA (N = 4, assay performed in duplicates). **(F)** Firefly-Luciferase-based cytotoxicity assay. T cell products were incubated with HER2+ (MDA-HER2) or HER2- (MDA) ffLUC-expressing target cells for 24h in combinations with BT. Target cell survival was determined based on luminescent signal (N = 4; assay was performed in duplicates). **(G)** Expansion rate of T cell products in rechallenge assay. T cells were counted every 3.5 days and re-plated at the starting density onto freshly coated HER2-Fc target proteins. Fold expansion relative to plating density is plotted. Data are mean ± SEM; *p < 0.05; **p < 0.01; ***p < 0.001 ; n.s., not significant.

First, we aimed to investigate whether CARs with universal recognition capability, upon binding HER2 through their specific linker, could induce an anti-tumor response similar to conventional HER2-specific CARs. The trastuzumab-derived recognition domain (9, 12) is replaced by the affinity-enhanced monomeric streptavidin 2 (mSA2) biotin-binding domain with its optimized hinge (7), thus we have ensured that the difference between the evolved anti-tumor effects depends solely on the recognition domains. Thus, we generated a UniCAR construct (mSA2.CD28.z) that consists of the same CD28 transmembrane, CD28 costimulatory and CD3ζ signal domain as the conventional HER2-specific CAR (4D5.CD28.z) (**Figure 1A**). Both vectors were packaged into RD114 pseudotyped retroviruses and transduced into primary human T cells.

Mean transduction efficiency in the CD3^+^ human lymphocyte population was 73.4% in the UniCAR and 84.0% in the HER2-CAR group, as judged by flow cytometry on day 4 post-transduction (**Figures 1B, C**). Immunoblot analysis confirmed the presence of UniCAR and HER2-CAR derived CD3ζ subunits in transduced T cells (**Supplementary Figure 2**).

### 3.2 UniCAR T cells targeted by biotinylated trastuzumab show similar *in vitro* activity and anti-tumor efficacy as conventional HER2-specific CAR T cells

*In vitro* activation, anti-tumor efficacy and long-term persistence of UniCAR T cells targeted by soluble biotinylated trastuzumab (BT) and HER2-CAR T cells targeted by a trastuzumab derived ScFv were investigated in activation, coculture and rechallenge experiments. First, in a cell-free activation assay, we confirmed that various concentrations of biotinylated trastuzumab could induce UniCAR T cell activation on plates coated with immobilized recombinant HER2-Fc chimera protein. From the amount of IFNγ secreted during 24h incubation, we concluded that in the presence of the HER2-specific soluble linker, UniCAR T cell activation was strictly antigen-dependent and correlated with the concentration of the linker. Without biotinylated trastuzumab, there was no cytokine secretion, confirming specificity (**Figure 1D**, green area). However, conventional HER2-CAR T cells at the same target concentration secreted higher levels of IFNγ than UniCAR T cells with the highest (10 µg/ml) BT concentration (**Figure 1D**, HER2-CAR). This maximal concentration of biotinylated trastuzumab could only induce limited UniCAR T cell activation in the absence of the HER2 target antigen, suggesting that soluble linkers alone have a weak capacity to organize UniCARs into supramolecular clusters (CAR synapse) and thereby initiate effector cell activation (**Figure 1D**, blue area). We confirmed that this background activation is due to mSA2 crosslinking by BT carrying more than one biotin per antibody molecule (data not presented). Non-transduced T cells (NT) were not activated even in the presence of high doses of the BT and the HER2 target (**Figure 1D**, NT).

In contrast to their subpar activity on panned molecular targets, in cocultures with trastuzumab-resistant HER2^+^ MDA-HER2 cells at a 1:1 effector to target ratio, biotinylated trastuzumab targeted UniCAR T cells secreted IFNγ at amounts comparable to conventional HER2-CAR T cells (**Figure 1E**; UniCAR+BT or HER2-CAR vs. all other samples: p < 0.001; UniCAR+BT vs. HER2-CAR: n.s.) and also induced killing of monolayer tumor cell cultures with similar potency (**Figure 1F**; UniCAR+BT or HER2-CAR vs. all other samples: p < 0.01; UniCAR+BT vs. HER2-CAR: n.s. and **Supplementary Figure 3**). There was no cytokine secretion or tumor lysis in the absence of target (**Figures 1E, F**, MDA), CAR (**Figures 1E, F**, NT) or BT (**Figures 1E, F**, UniCAR).

Having explored the early activation and anti-tumor efficacy of UniCAR and conventional HER2-CAR T cells, we examined whether the indirect or direct HER2 binding impacts the long-term *in vitro* persistence of the CAR T cells. UniCAR T cells in the presence of 10 µg/ml biotinylated trastuzumab and, for comparison, HER2-CAR T cells were restimulated twice weekly on immobilized HER2-Fc molecules. NT T cells with or without BT and UniCAR T cells without BT served as controls. We found that the proliferative capacity of BT-targeted UniCAR T cells was comparable, although slightly lower than that of conventional HER2-CAR Ts (**Figure 1G**).

### 3.3 UniCAR T cells targeted by biotinylated trastuzumab recognize and penetrate three-dimensional trastuzumab-resistant MDA-HER2 tumor spheroids

**Figure 2:**
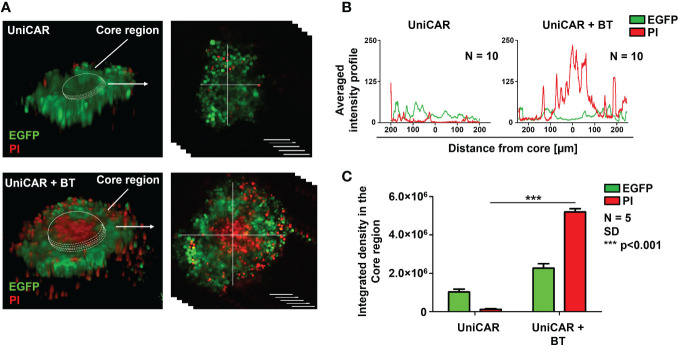
UniCAR T cells infiltrate trastuzumab-resistant tumor spheroids in the presence of biotinylated trastuzumab linker. **(A)** Representative images (at 24h) for detecting the cytolytic activity of UniCAR T cells ± 10 µg/ml BT against MDA-HER2.ffLUC (eGFP tagged; green) spheroids. Dead cells were visualized by PI uptake (red). **(B)** Histograms represent the averaged intensity profiles of eGFP and PI signals in cross-sections of spheroids (N =10). **(C)** Integrated intensities of eGFP (green) and PI (red) signals. Histograms show mean ± SEM; N = 5;***p < 0.001.

We have previously demonstrated that the MUC4 and CD44-rich extracellular matrix (ECM) in trastuzumab-resistant tumors, as in MDA-HER2, develops an impenetrable barrier to antibodies and thus effectively inhibits trastuzumab-dependent NK cell-mediated cytotoxicity. At the same time, conventional HER2-CAR T cells, as actively moving cell therapeutics, recognize the ECM-masked HER2 target antigen in the spheroid core and induce a potent cytotoxic response (9). The finding that BT-targeted UniCAR T cells recognized and killed MDA-HER2 in monolayer cultures encouraged us to investigate their anti-tumor functions in tumor spheroids with an established ECM. To explore whether UniCAR T cells can access ECM-masked HER2^+^ cells through a soluble HER2-targeting linker, we set up a coculture experiment in which MDA-HER2 spheroids were cultured with effector UniCAR T cells for 24h in the presence or absence of biotinylated trastuzumab. Using confocal microscopy (16), we visualized (**Figure 2A**) and analyzed (**Figures 2B, C**) tumor cell killing indicated by incorporation of propidium iodide (PI). UniCAR T cells in the presence of 10 µg/ml biotinylated trastuzumab showed substantially higher cytolytic activity in the inner spheroid core than in the absence of the BT linker (p < 0.001) (**Figure 2C**).

### 3.4 Soluble biotinylated trastuzumab binds to the mSA2 domain immediately upon UniCAR T cell administration

**Figure 3:**
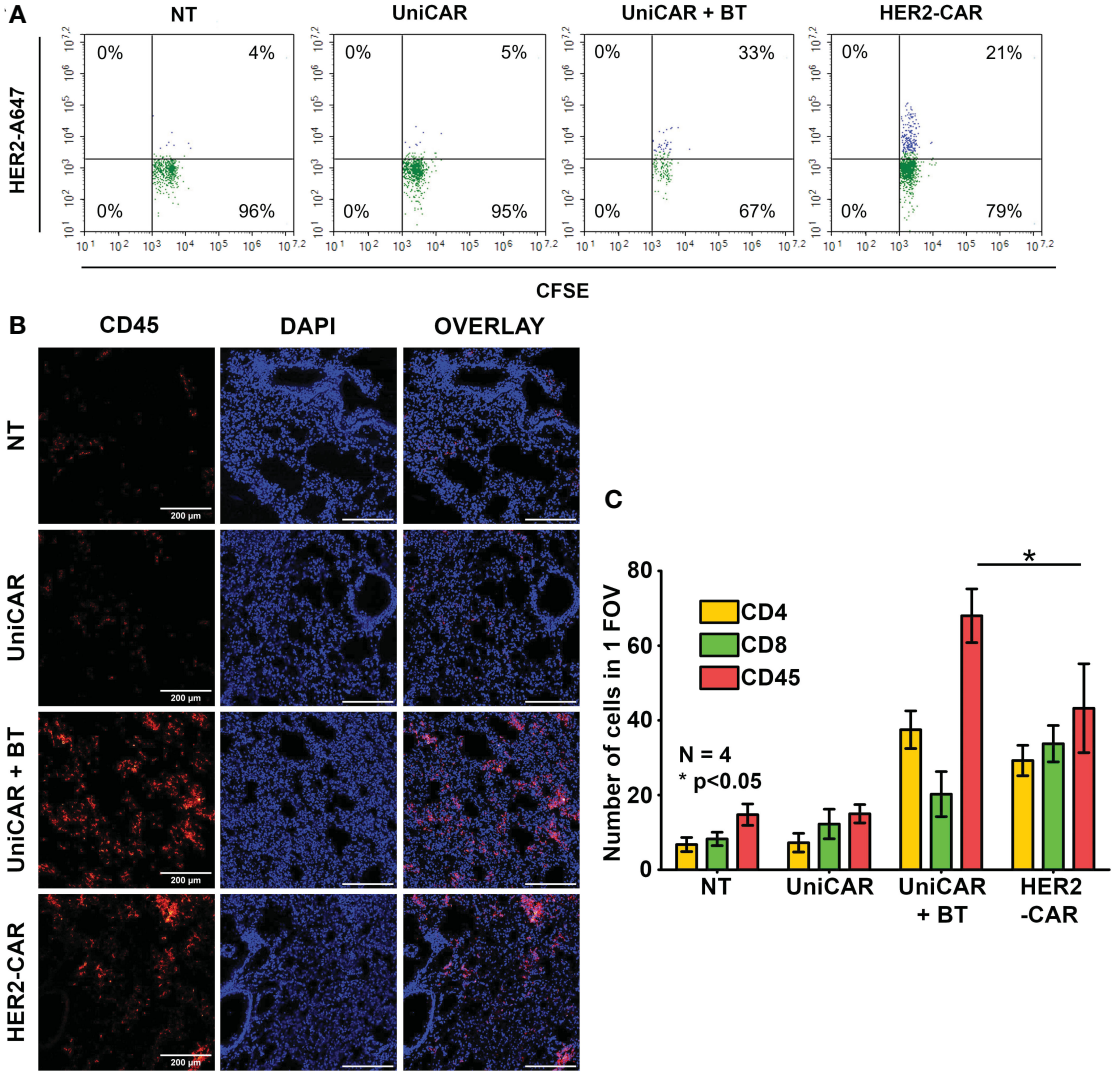
Generation of HER2-directed UniCAR T cells *in vivo.* Mice received a single i.v. dose of 20 × 10^6^ non-transduced (NT), UniCAR or HER2-CAR T cells. The UniCAR+BT mouse received 100 µg biotinylated trastuzumab in 100 µl PBS 15h prior T cell injection. **(A)** Representative flow cytometry histogram of A647-HER2 recognizing cells. **(B, C)** Representative images and summary data of frozen sections of the treated lungs 2 minutes after effector cell injection. **(B)** Excised lungs were fluorescently labeled for CD45 (red), and DNA (blue). **(C)** Mean cell number in 1 field of view (FOV). Histograms show mean ± SEM; N = 4; *p < 0.05.

Given that UniCAR T cells alone do not penetrate spheroids (**Figure 2**) and trastuzumab alone does not penetrate these spheroids (9), we hypothesized that for effective tumor penetration UniCAR T cells and BT must pre-assemble. To reveal whether this pre-assembly happens in the therapeutic setting, in our first *in vivo* experiment 20x10^6^ CFSE prelabeled UniCAR T cells were administered i.v. into an NSG mouse pretreated i.p. with 100 µg of biotinylated trastuzumab 15 h before effector cell injection. A blood sample was taken two minutes after tail vein injection, and the presence of biotinylated trastuzumab-bound UniCARs in the T cell membrane was determined using the Alexa Fluor 647-conjugated HER2 (A647-HER2). Conventional HER2-CAR T cells served as positive, NT and UniCAR T cells without BT pretreatment as negative controls. We found that almost immediately after UniCAR T cell injection, soluble BT bound to the mSA2 domain, as indicated by A647-HER2 binding to the CFSE^+^ population, similarly to the HER2-CAR positive control (**Figure 3A**, UniCAR + BT: 33% vs. HER2-CAR: 21%). We confirmed that CFSE-positive cells are identical to the human CD45-positive cells (**Supplementary Figure 4**, upper panel, second row, CD45-APC vs. CFSE). However, we could recover considerably fewer human T cells from the UniCAR + BT mouse than from the other animals by sampling the same volume. Since upon CAR T cell injection, effector cells primarily gather in the lungs, with a notable presence in other organs beginning only 14 hours post-injection (20), in search of a possible explanation we have investigated the presence of injected cells in the lungs and found an significantly increased density of CD45-positive human T cells in the lungs of the UniCAR + BT mouse compared to the other animals (**Figures 3B, C**, red columns). We observed a predominance of CD4+ T cells in the lungs of the UniCAR T + BT-treated mouse, whereas a balance of CD4+ and CD8+ T cells was observed in the lungs of HER2-CAR T cell injected animal. (**Figure 3C**, yellow and green columns and **Supplementary Figure 5**). Interestingly, A647-HER2 binding was also observed in a CFSE^-^/CD45^-^ population of UniCAR + BT treated mice, indicating the presence of mouse-derived, Fc-receptor expressing phagocytes that can bind trastuzumab (**Supplementary Figure 4**, bottom panel).

### 3.5 UniCAR T cells targeted by biotinylated trastuzumab induce unexpected death of the treated animals

**Figure 4:**
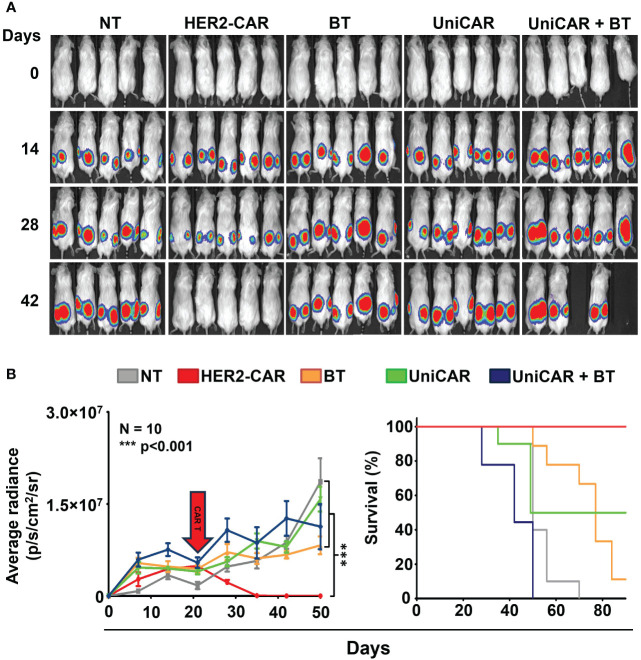
Toxicity in animals co-treated with UniCAR T cells and biotinylated trastuzumab. Mice were injected s.c. with 3 × 10^6^ MDA-HER2.ffLUC cells to establish tumor xenografts, then they received a single i.v. dose of 2 × 10^6^ T cells on day 21 (red arrow). Mice (co-)treated with biotinylated trastuzumab received 100 µg BT in 100 µl PBS i.p. twice weekly from day 21. Tumor growth was followed by bioluminescence imaging. **(A)** Representative images of MDA-HER2.ffLUC injected animals. Images were taken 10 minutes after i.p. injecting 100 µg D-luciferin in 100 µL PBS by an IVIS Spectrum CT instrument. (**B** left panel) Quantitative bioluminescence imaging data of MDA-HER2.ffLUC xenografts (radiance = photons/s/cm2/sr; HER2-CAR vs. all other treatments: ***p < 0.001). (**B** right panel) Kaplan-Meier survival curve (HER2-CAR vs. all other treatments: ***p < 0.001).

To compare the *in vivo* anti-tumor effect of UniCAR and conventional CAR T cells, subcutaneous HER2^+^ MDA-HER2.ffLUC xenografts were grown in NSG mice and treated with a single i.v. dose of 2 × 10^6^ UniCAR or HER2-CAR T cells on day 21 post tumor cell inoculation (**Figure 4A**, **Supplementary Figure 1**). Except for the control group for off-target effects (UniCAR group), animals treated with UniCAR T cells also received 100 µg biotinylated trastuzumab in 100µl PBS i.p. on the day of CAR T cell injection and twice weekly thereafter (UniCAR+BT group). As the *in vivo* binding experiment (**Supplementary Figure 3**, bottom panel), our previous results (9) and the experience of others (21)have shown that NSG mice may have functional monocytes and macrophages, we investigated the potential antibody-dependent cellular cytotoxicity (ADCC) induced by biotinylated trastuzumab treatment in a linker only group (BT group). Mice treated with non-transduced T cells served as a reference group (NT group) (**Supplementary Figure 1**).

Surprisingly, mice co-treated with UniCAR T cells and biotinylated trastuzumab started to die within a week of CAR T injection; moreover, all the animals were lost within four weeks, with no appreciable tumor regression. No similar effect was seen in any of the control groups. As expected from earlier experience, all the mice in the HER2-CAR T cell treated group were cured (**Figures 4A, B**).

### 3.6 UniCAR + BT treatment resulted in on-target-off tumor toxicity in the lungs

**Figure 5:**
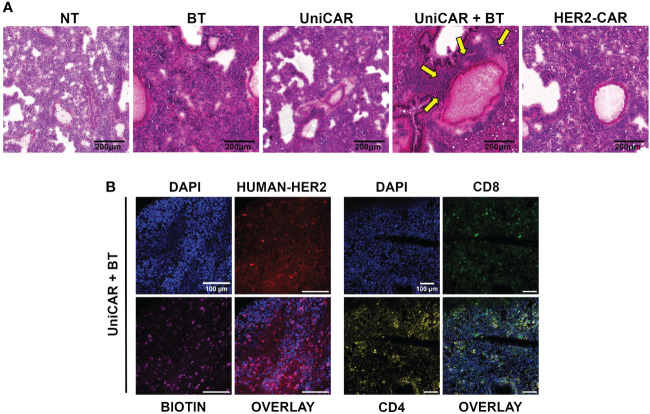
On target off tumor toxicity in the lungs of animals co-treated with UniCAR T cells and biotinylated trastuzumab. **(A)** Hematoxylin-eosin-stained sections from lung tissues of mice at 27 (UniCAR+BT) or 64 days (NT; HER2-CAR; BT; UniCAR) after effector cell injection. Arrows indicate massive cellular infiltration around the vessels. **(B)** Representative frozen section of a UniCAR+BT co-treated lung 27 days after effector cell injection. Excised lungs were fluorescently labeled for HER2 (red), biotin (magenta), CD8+ human T cells (green), CD4+ human T cells (yellow), and DNA (blue).

Routine pathological H&E stained tissue sections of the treated animals revealed a massive cellular infiltration around the blood vessels in the lungs of mice co-treated with UniCAR T cells and biotinylated trastuzumab (**Figure 5A**; UniCAR+BT, yellow arrows). No abnormalities were found in the liver, spleen, kidneys, or heart (**Supplementary Figure 6**).

Subsequently, a systematic immunohistochemical screen revealed biotin accumulating and trastuzumab binding cells in all treatment groups (**Supplementary Figure 7**). In addition, in the lungs of UniCAR+BT treated mice we detected many CD4^+^ and CD8^+^ human lymphocytes on day 27 post-treatment, indicating the presence of UniCAR T cells (**Figure 5B**, **Supplementary Figure 7**). We could also see human CD8^+^ lymphocytes in the lungs of NT, UniCAR and HER2-CAR T cell treated animals on day 64 post effector cell injection, indicating the emergence of xeno recognition (**Supplementary Figure 7**; NT, UniCAR, HER2-CAR). However, in the lungs of UniCAR+BT-treated animals, the cellular infiltration and pathological effects appear to be mainly due to the presence of murine macrophages, which are detectable in high numbers along with human T cells only in these animals (**Supplementary Figure 8**; UniCAR+BT). In contrast, in the lungs of HER2-CAR-treated mice, less intensive mouse macrophage infiltration was observed even at day 64 post effector cell injection, whereas the number of mouse neutrophil granulocytes was slightly increased (**Supplementary Figure 8**; HER2-CAR). Less intensive macrophage or granulocyte infiltration was observed in the lungs of UniCAR-treated mice if no BT was present (**Supplementary Figure 8**; UniCAR).

### 3.7 UniCAR T cells targeted by biotinylated trastuzumab infiltrate HER2-positive xenografts *in vivo*

**Figure 6:**
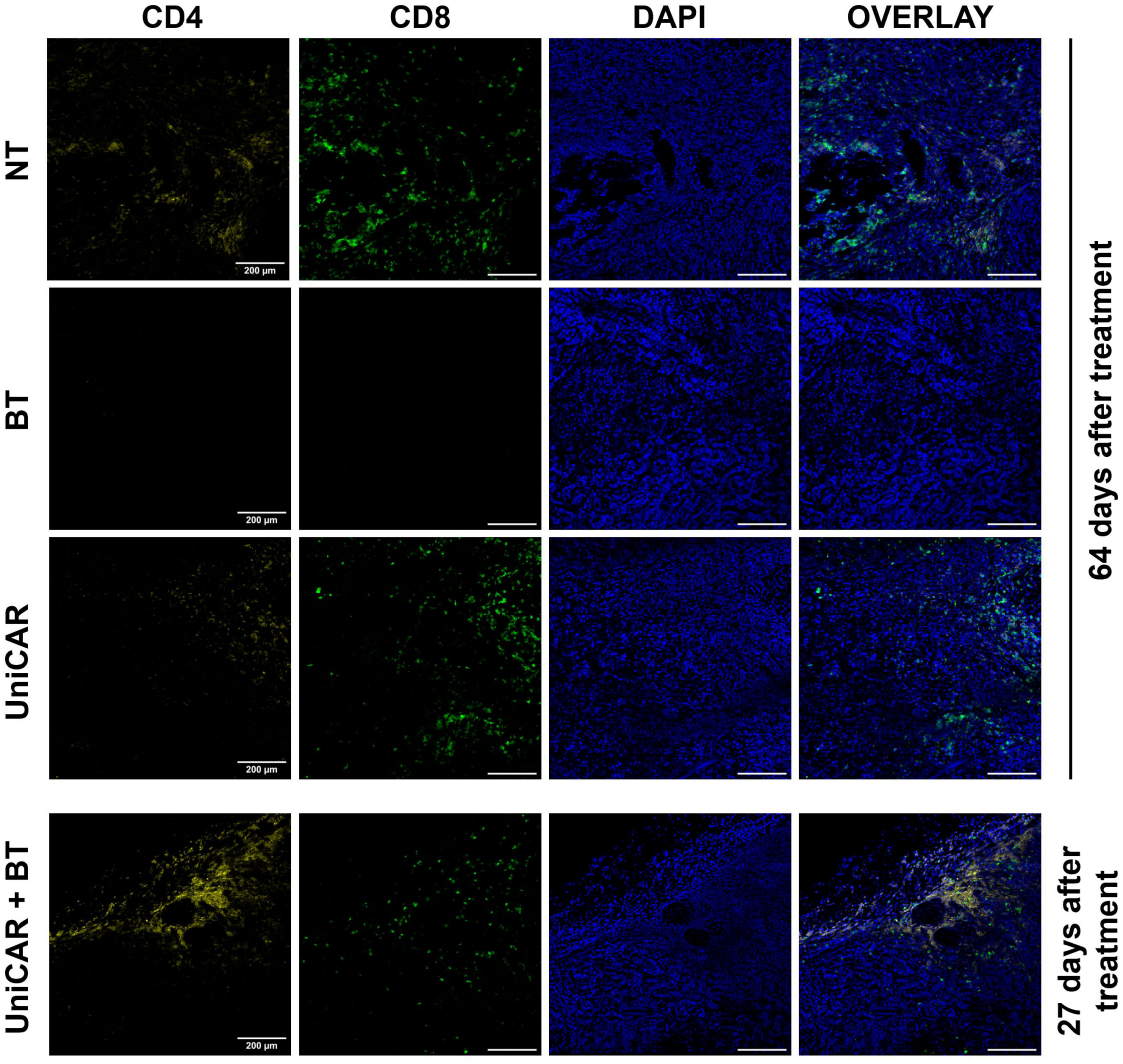
Tumors from mice co-treated with UniCAR T cells and biotinylated trastuzumab show massive CD4^+^ T cell infiltration. Frozen sections of excised MDA-HER2.ffLUC tumors were fluorescently labeled for human CD4^+^ (yellow) or CD8^+^ T cells (green), and DNA (blue) on day 27 (UniCAR+BT) or day 64 (NT; BT; UniCAR) after effector cell injection.

In tumors co-treated with UniCAR T cells and BT, we detected a high number of infiltrating human T lymphocytes as early as 27 days after inoculation (**Figure 6**, UniCAR+BT). We confirmed that the majority of tumor-infiltrating lymphocytes were CD4^+^ T cells. In contrast, in mice treated with UniCAR T cells only or with NT T cells only, on day 64 post effector cell injection tumors were mainly infiltrated with CD8^+^ T cells, which have possibly proliferated due to allorecognition of the human tumor tissue (**Figure 6**, UniCAR, NT). In the HER2-CAR-treated group, the rapid cytolytic effect did not allow tumor excision. Overall, these results demonstrate that UniCAR T cells coupled with biotinylated-trastuzumab can penetrate trastuzumab-resistant xenografts.

## 4 Discussion

In recent years, the development and optimization of CAR T cell-based therapeutic systems with universal recognition capabilities has become increasingly popular. In contrast to the single-antigen binding property of conventional CAR T cells, these UniCAR T cells offer the recognition of versatile antigens using a therapeutic cell product expressing a single CAR species. This could lead to breakthroughs in treating solid tumors with high antigen heterogeneity (5–8, 22–24). However, it should be noted that, in addition to their many positive features, UniCAR systems also have certain limitations. Instead of the direct antigen binding based recognition of conventional CAR T cells, these effectors indirectly recognize the target through a linker molecule that may affect the stability of the CAR immune synapse, thus limiting the avidity of these reprogrammed T cells (25). In addition, in solid tumor microenvironments where the extracellular matrix inhibits access of small molecules such as therapeutic antibodies (10) thereby precluding efficient antibody-mediated cytotoxicity through NK cells (9), soluble linker molecules may also be unable to activate UniCAR T cells. Moreover, it has not been investigated whether the mechanism of action of UniCAR systems is different from that of monoclonal antibodies, whereby the soluble antibody first binds to the tumor cell, and then this antibody-coated tumor cell recruits immune cells through the Fc region of the mAb (26).

Therefore, in the present work, we have explored the sequence of *in vivo* molecular events and investigated whether UniCAR T cells that recognize the target antigen through a soluble linker molecule are also inhibited by the rich extracellular matrix of CD44^+^ tumors in the same way as NK cells recognizing HER2-bound trastuzumab, or whether they can efficiently recognize the HER2 target by penetrating the matrix in a similar way to conventional CAR T cells.

We successfully generated CAR T cells expressing an affinity-enhanced monomeric streptavidin 2-derived (mSA2) universal recognition unit. The transduction efficiency of the UniCAR was identical to conventional CARs and in non-cellular activation assays and coculture experiments these UniCAR T cells were efficiently activated and induced specific anti-tumor responses in the presence of a HER2-recognizing linker and molecular or membrane-bound HER2 target. We also confirmed that the activation was proportional to the linker concentration. This result resonates well with the recent findings of Ruffo and colleagues, where the killing kinetics of effector cells in a similar linker-based UniCAR system showed a similar trend (8).

We then examined whether spheroids developed from a trastuzumab-resistant MDA-HER2 cell line could be efficiently eliminated by UniCAR T cells in the presence of the linker molecule. Our results demonstrate that UniCAR T cells guided by biotinylated trastuzumab efficiently penetrate the spheroid and induce cell death in its core region. The picture is comparable to our previous results, in which conventional HER2-CAR T cells showed the same efficacy against trastuzumab-resistant spheroids (9).

Biotin-avidin interactions generally form instantly and are more stable than antibody-antigen interactions, which can vary widely in their binding kinetics and affinity. While biotin-avidin is one of the strongest non-covalent biological interactions known, with a dissociation constant (Kd) in the range of 10^-15^ M (27), the strength of antibody-antigen interactions can vary widely, typically with a dissociation constant (Kd) ranging from 10^-6^ to 10^-12^ M (28). This extremely high affinity makes biotin-avidin binding practically irreversible under physiological conditions. Based on these, we hypothesized that this stable and rapid association might impact effector functions. In our *in vivo* model, we could verify that upon effector cell administration, the mSA2 domain of UniCAR constructs immediately engages the soluble biotinylated trastuzumab linker that circulates in the blood. We have thus demonstrated that in contrast to mAb-based therapies, where the primary and initial target of the antibody is the tumor cell, not the effector cell that expresses the Fc receptor (26), UniCAR T cells bind to the linker before encountering the target and thus behave as conventional CAR T cells. This observation is also consistent with the efficient killing of spheroid tumors by BT-guided UniCAR T cells, but not by UniCAR T cells without BT, particularly in light of earlier observations that trastuzumab alone does not penetrate spheroids on the same time scale (9).

From our present observations, we conclude that the UniCAR concept is only viable if the molecular interaction between the receptor and the linker is a high-affinity bond. Only in such a case can a condition be established in which assembled, fully armed CAR T cells do not need to rely on the binding equilibrium shifted towards association by the high local concentration of soluble linker molecules. This is particularly important since, due to diffusion limitations, the concentration of the soluble linker in the TME can be very low in the case of solid tumors with a well developed ECM.

In order to confirm that UniCAR T cells targeted by a HER2-specific soluble linker can penetrate established tumor xenografts, we performed an *in vivo* experiment in which mice were xenotransplanted with trastuzumab-resistant MDA-HER2 cells. We allowed the extracellular matrix to build up for 21 days, and then the animals were treated with CAR T cells i.v. and biotinylated trastuzumab i.p. The clinical significance of this methodology lies in allowing the linker-UniCAR interaction to occur exclusively in the treated animals following injections, which can thus be modulated by the biodistribution of cells and linker molecules (6, 29). This differs from *in vitro* experiments, where the two components could interact immediately.

Using this model, we observed a high level of BT-guided UniCAR T cell infiltration in the tumors upon termination of the experiment. Non transduced T cells and UniCAR T cells in the absence of BT also penetrated the tumors, however, these tumors have shown a dominance of CD8^+^ T cells, indicating an allogeneic reaction which maintained T cell expansion up to termination on day 64 post inoculation. In contrast, BT-guided UniCAR T cells exhibited CD4^+^ phenotype more frequently than CD8^+^ phenotype, clearly indicating the specific interaction linking the UniCAR to the HER2 target through biotinylated trastuzumab. Since both CD4^+^ and CD8^+^ UniCAR T lymphocytes can establish this specific interaction, a balanced presence of these species is expected based on earlier experiments with HER2 specific conventional CARs (9, 12, 30). We speculate that this balance may be shifted to CD4 positivity over the 4 weeks of treatment owed to preferred recruitment of xenoreactive CD8^+^ T cells to other organs. Furthermore, histopathological analysis of lung sections obtained from UniCAR+BT treated mice revealed human CD4^+^ and CD8^+^ lymphocyte infiltration in the vicinity of biotin and/or HER2-positive cells and this off-tumor on-target effect may have sequestered a part of the CD8^+^ fraction of the therapeutic cell product, causing a consequent CD4^+^ accumulation in the tumor.

Importantly, the specific dual targeting of HER2 and native biotin has also led to a rapidly escalating immune response localized to the lung. This was initiated by biotinylated trastuzumab and UniCAR T cells but escalated by the mouse mononuclear phagocyte system, partly through their recognition of human Fc in BT (31), and partly through recruitment of both macrophages (32) and neutrophils (33) by T-cell derived type 2 cytokines. This finding is supported by the significant number of mouse-derived A647-HER2-bound cells detected in the blood of UniCAR+BT co-treated mice. However, the large number of human lymphocytes, predominantly CD4^+^ UniCAR T cells, that appeared in the lungs immediately after the injection and persisted there may also have played a role in the development of lethal side effects. These cells have recently been shown to play a key role in maintaining long-term responses, but also to have a higher toxic potential (34). This massive multidirectional reaction has led to the demise of mice before tumors could be eradicated. However, from this observation, we can also draw the general conclusion that following target recognition in HER2-expressing tissues, biotinylated-trastuzumab-bound UniCAR T cells are activated and proliferate. CARs are recycled and resynthesized in daughter cells during clonal expansion. Consequently, the actual local tissue concentration of biotinylated-trastuzumab determines whether effector cells bind the ligand through BT, or in the relative absence of BT, the native biotin present in the tissues.

The present results demonstrate that universal CAR systems based on soluble linker molecules can be an effective alternative to conventional CAR T cells, even in solid tumors with a well-developed extracellular matrix. Here, we provide evidence that UniCAR T cells are well suited for use in patients who have become resistant to antibody therapy. However, we also show that the correct choice of recognition unit-linker pair is critical in terms of both therapeutic efficacy and safety; a linker which allows its stable pre-assembly with the CAR, but not present even partially in the treated organism is needed.

## Data availability statement

The original contributions presented in the study are included in the article/**Supplementary Material**, further inquiries can be directed to the corresponding author/s.

## Ethics statement

The studies involving humans were approved by Regional and Institutional Committee for Research Ethics (RKEB.5378/2019), University of Debrecen. The studies were conducted in accordance with the local legislation and institutional requirements. Written informed consent for participation was not required from the participants or the participants’ legal guardians/next of kin in accordance with the national legislation and institutional requirements. The animal study was approved by National Ethical Committee for Animal Research (# 5-1/2018/DEMÁB), University of Debrecen. The study was conducted in accordance with the local legislation and institutional requirements.

## Author contributions

LN: Data curation, Methodology, Project administration, Validation, Visualization, Writing – original draft, Writing – review & editing, Investigation. MM-C: Methodology, Writing – original draft, Writing – review & editing, Investigation. IR: Methodology, Writing – original draft, Writing – review & editing, Investigation. GV: Conceptualization, Funding acquisition, Methodology, Project administration, Supervision, Writing – original draft, Writing – review & editing. ÁS: Conceptualization, Funding acquisition, Investigation, Methodology, Project administration, Resources, Supervision, Validation, Visualization, Writing – original draft, Writing – review & editing.

## Supplementary material

Supplementary Figure 1 Schedule and applied treatments of the *in vivo* mouse experiment. A total of 25 female NOD NOD.Cg-Prkdcscid/Il2rgtm1Wjl/SzJ mice received 2 × 10^6^ MDA-HER2.ffLUC cells subcutaneously in 100µl PBS and 100µl Matrigel. Effector cell treated mice received on day 21 (red arrow) a single i.v. dose of 2 × 10^6^ NT T cells (NT T cell, grey) or HER2-CAR T cells (HER2-CAR, red), or UniCAR T cells (UniCAR, green). Biotinylated trastuzumab (BT, orange) and UniCAR plus biotinylated trastuzumab (UniCAR+BT, blue) treated animals trastuzumab received 100 µg biotinylated trastuzumab in 100 µl PBS i.p. twice a week during the whole experiment, starting from day 21 as well. Tumor growth was followed by bioluminescence imaging.

Supplementary Figure 2 Expression of HER2-CAR and UniCAR confirmed by Western Blot analysis. Western Blot was performed from 2 × 10^6^ non-transduced (NT), HER2-CAR-transduced (HER2 CAR) and UniCAR-transduced (UniCAR) human T cells. Human CD3ζ was probed with mouse anti-human CD3ζ antibody at 1μg/ml. HRP-conjugated anti-mouse IgG antibody served as secondary antibody. Actin was used as a positive control.

Supplementary Figure 3 Conventional light microscopy images of the formation of activation clumps. UniCAR +/- 10 µg/ml BT or HER2-CAR or NT T cells +/- 10 µg/ml BT were cocultured with HER2- (MDA) or HER2+ (MDA-HER2) target cells for 24 hours. Images were taken with a Zeiss LSM 880 laser scanning microscope using the non-descanned transmitted light detector and processed with Fiji ImageJ 1.53t.

Supplementary Figure 4 Flow cytometric analysis of human T cell products recovered from mouse blood samples after effector cell injection. All human derived T cell products were labeled with CFSE prior i.v. injection of 20 × 10^6^ T cells. Blood samples were stained with anti-human CD45-APC and a A647-HER2 conjugate in two separate aliquots.

Supplementary Figure 5 Distribution of human T cell phenotypes in mouse lung upon T cell injection. Representative frozen sections of mice lungs immediately upon effector cell injection. Samples were stained for human CD8 (green), human CD4 (yellow) and DNA (DAPI, blue) to detect human cytotoxic and helper T lymphocytes. Images were taken with Zeiss LSM880 confocal fluorescence microscope. Image processing was done with Fiji ImageJ 1.53t software. Scalebar = 200µm.

Supplementary Figure 6 Comparative table of all excised mouse organs stained with H&E. Staining was carried out with a standard H&E protocol. Samples were scanned with a Pannoramic Confocal digital slide scanner (3DHISTECH, Budapest, Hungary) in transmission mode. Images were processed with CaseViewer software (3DHISTECH). Yellow arrows showing cell infiltration along blood vessels in the lung of mice treated with UniCAR T cells and biotinylated trastuzumab.

Supplementary Figure 7 Fluorescent images of all lung samples probed for CD8, CD4, biotin and human HER2. Representative frozen sections of mice lungs 27 days (UniCAR + BT) or 64 days (all other groups) after effector cell injection. The CD8/CD4 panel (left) shows human CD8+ cytotoxic T cells (green) and human CD4+ helper T cells (yellow) infiltrated in the lung. The biotin/human HER2 panel shows native biotin (magenta) and HER2 in the lung. Images were taken with a Zeiss LSM880 confocal fluorescence microscope. Image processing was done with Fiji ImageJ 1.53t software.

Supplementary Figure 8 Comparative table of all mouse neutrophile granulocyte and mouse macrophage micrographs from the lung samples. Images were taken with a Zeiss LSM880 confocal fluorescence microscope. Images show the presence of mouse neutrophil granulocytes (cyan) and mouse macrophages (orange) Image postprocessing was done with Fiji ImageJ software. (orange). Image processing was done with Fiji ImageJ 1.53t software.
