# Supplementary figures and images for: Universal CAR T cells targeted to HER2 with a biotin-trastuzumab soluble linker penetrate spheroids and large tumor xenografts that are inherently resistant to trastuzumab mediated ADCC
Source: Front Immunol. 2024 Mar 18;15:1365172. doi: 10.3389/fimmu.2024.1365172 (PMC10982377; doi:10.3389/fimmu.2024.1365172)

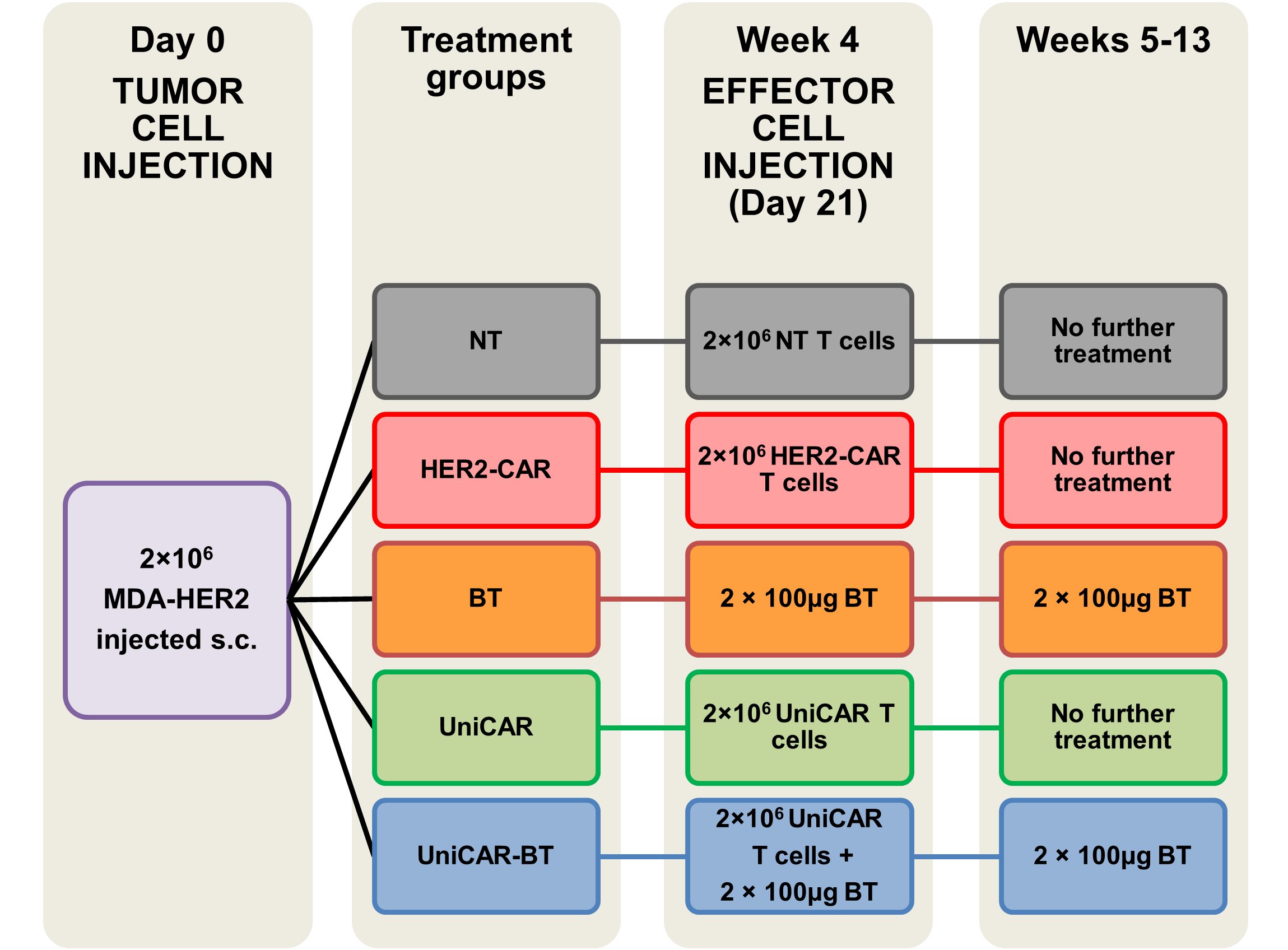

Supplement: Supplementary Figure 1 — Schedule and applied treatments of the in vivo mouse experiment. A total of 25 female NOD NOD.Cg-Prkdcscid/Il2rgtm1Wjl/SzJ mice received 2 × 10^6^ MDA-HER2.ffLUC cells subcutaneously in 100µl PBS and 100µl Matrigel. Effector cell treated mice received on day 21 (red arrow) a single i.v. dose of 2 × 10^6^ NT T cells (NT T cell, grey) or HER2-CAR T cells (HER2-CAR, red), or UniCAR T cells (UniCAR, green). Biotinylated trastuzumab (BT, orange) and UniCAR plus biotinylated trastuzumab (UniCAR+BT, blue) treated animals trastuzumab received 100 µg biotinylated trastuzumab in 100 µl PBS i.p. twice a week during the whole experiment, starting from day 21 as well. Tumor growth was followed by bioluminescence imaging. [file Image_1.jpeg]

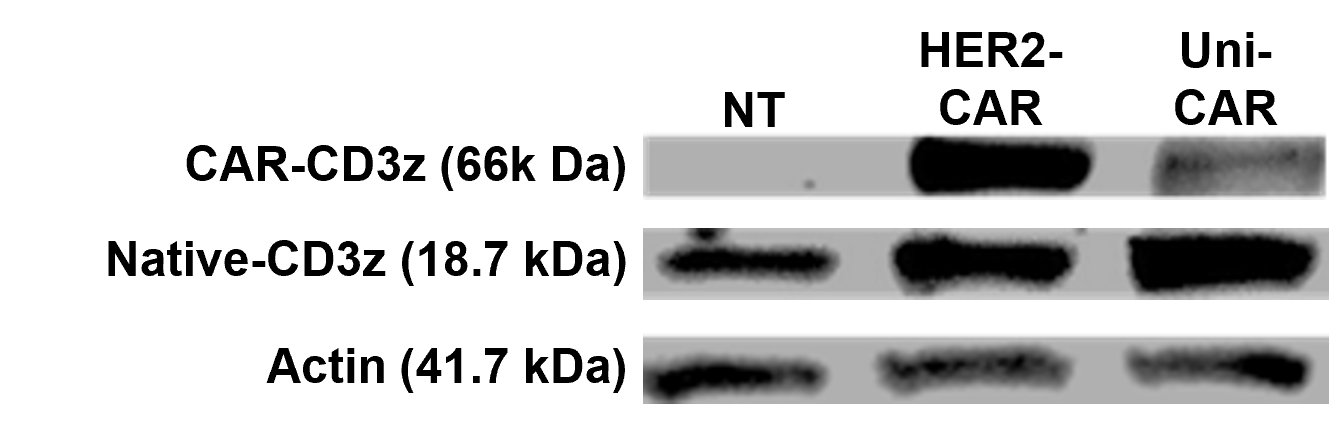

Supplement: Supplementary Figure 2 — Expression of HER2-CAR and UniCAR confirmed by Western Blot analysis. Western Blot was performed from 2 × 10^6^ non-transduced (NT), HER2-CAR-transduced (HER2 CAR) and UniCAR-transduced (UniCAR) human T cells. Human CD3ζ was probed with mouse anti-human CD3ζ antibody at 1μg/ml. HRP-conjugated anti-mouse IgG antibody served as secondary antibody. Actin was used as a positive control. [file Image_2.jpeg]

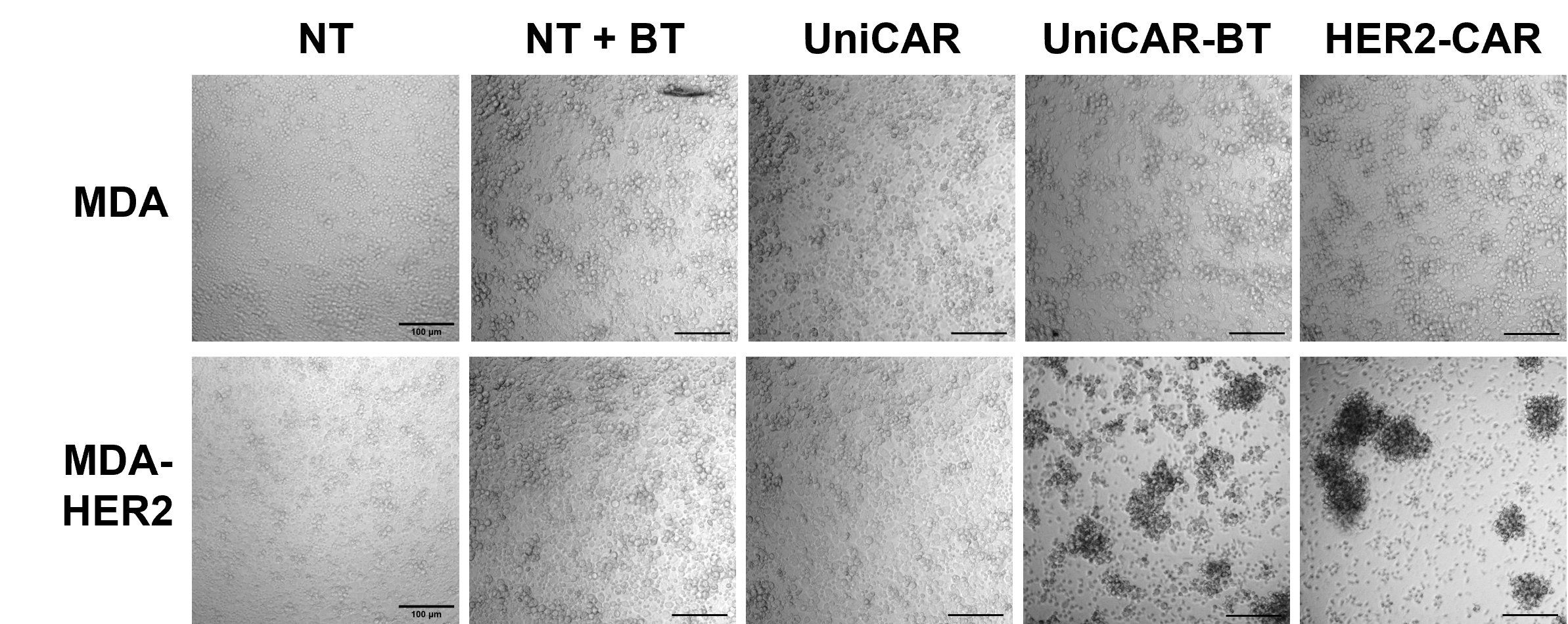

Supplement: Supplementary Figure 3 — Conventional light microscopy images of the formation of activation clumps. UniCAR +/- 10 µg/ml BT or HER2-CAR or NT T cells +/- 10 µg/ml BT were cocultured with HER2- (MDA) or HER2+ (MDA-HER2) target cells for 24 hours. Images were taken with a Zeiss LSM 880 laser scanning microscope using the non-descanned transmitted light detector and processed with Fiji ImageJ 1.53t. [file Image_3.jpeg]

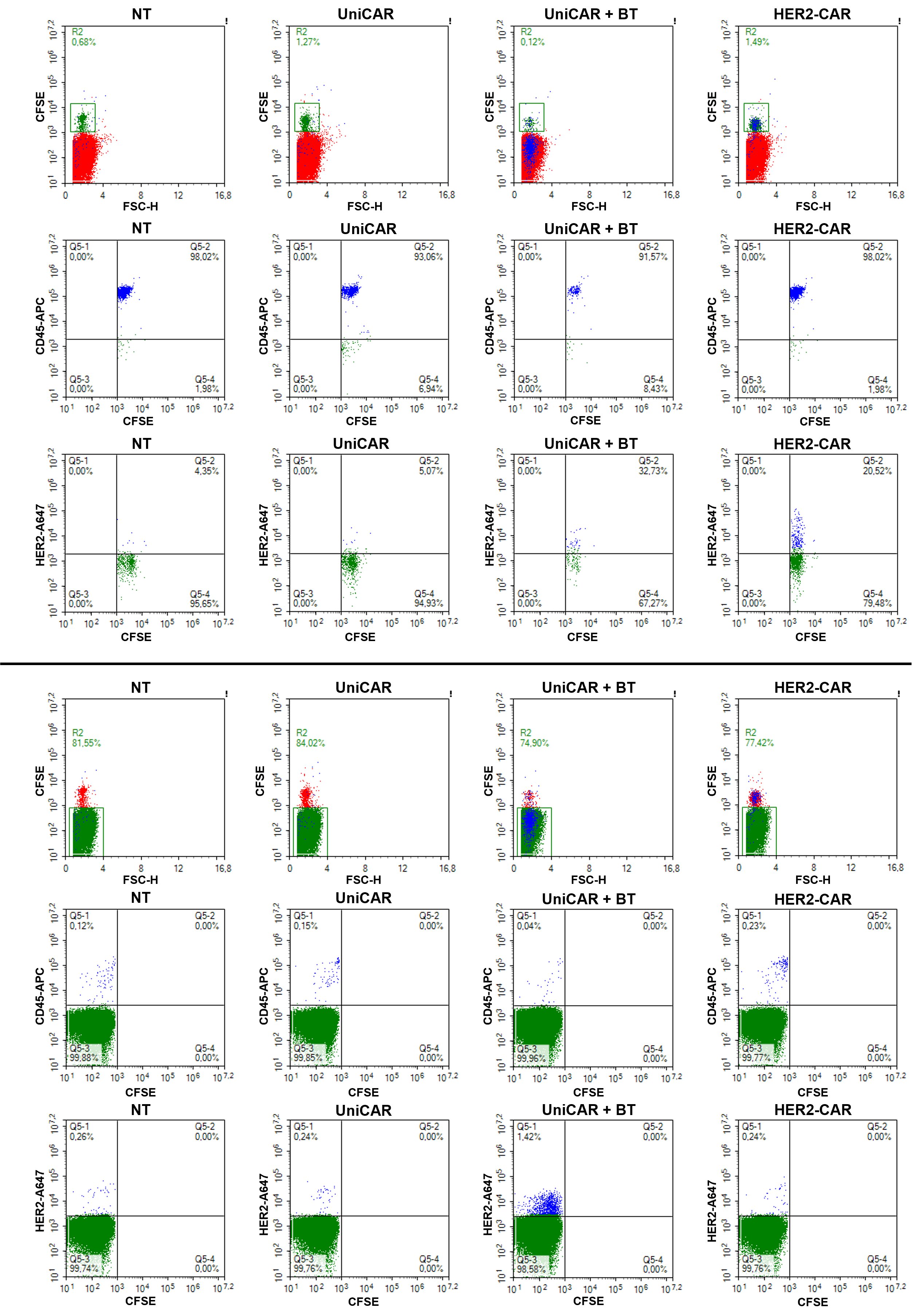

Supplement: Supplementary Figure 4 — Flow cytometric analysis of human T cell products recovered from mouse blood samples after effector cell injection. All human derived T cell products were labeled with CFSE prior i.v. injection of 20 × 106 T cells. Blood samples were stained with anti-human CD45-APC and a A647-HER2 conjugate in two separate aliquots. [file Image_4.jpeg]

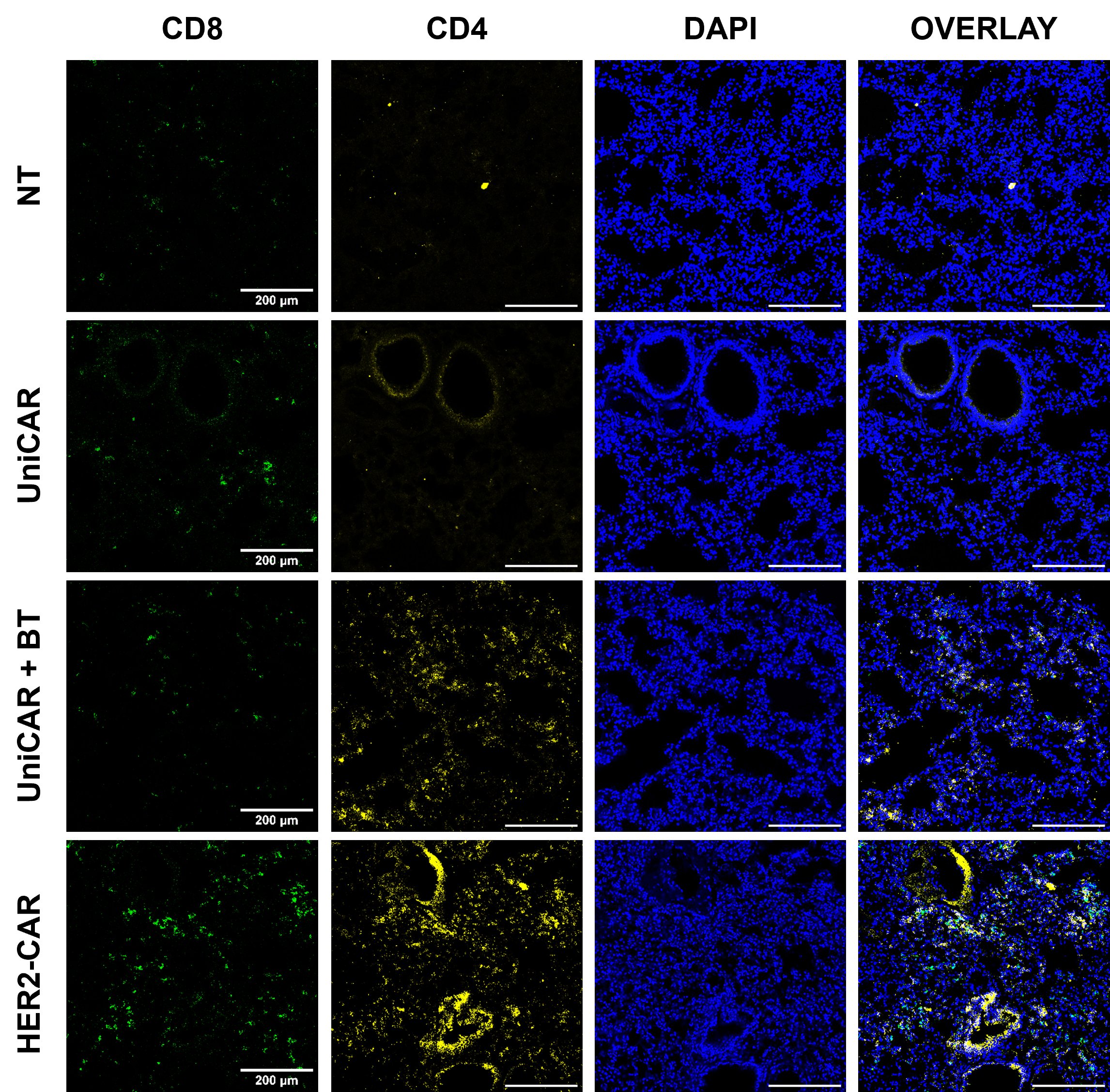

Supplement: Supplementary Figure 5 — Distribution of human T cell phenotypes in mouse lung upon T cell injection. Representative frozen sections of mice lungs immediately upon effector cell injection. Samples were stained for human CD8 (green), human CD4 (yellow) and DNA (DAPI, blue) to detect human cytotoxic and helper T lymphocytes. Images were taken with Zeiss LSM880 confocal fluorescence microscope. Image processing was done with Fiji ImageJ 1.53t software. Scalebar = 200µm. [file Image_5.jpeg]

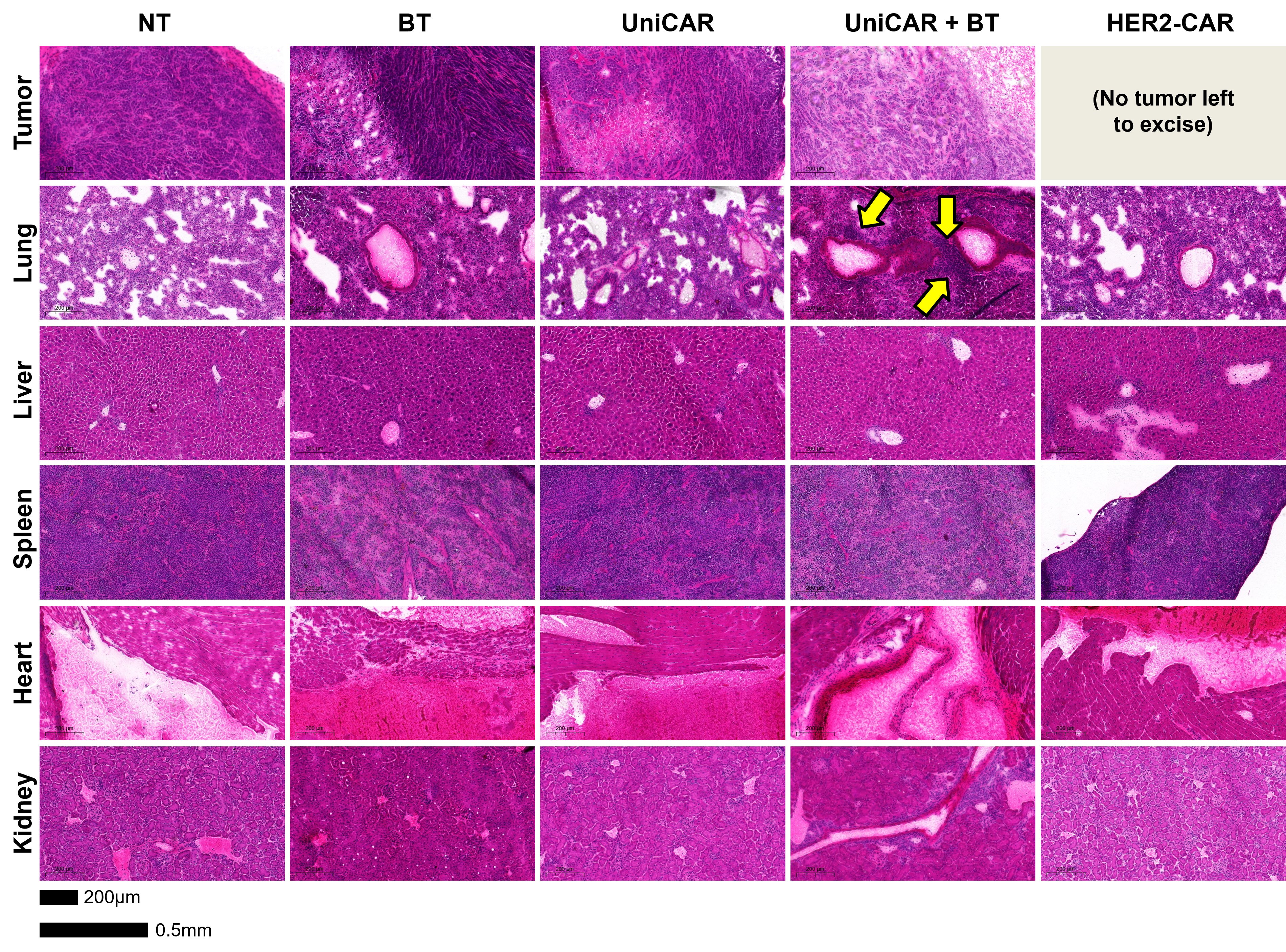

Supplement: Supplementary Figure 6 — Comparative table of all excised mouse organs stained with H&E. Staining was carried out with a standard H&E protocol. Samples were scanned with a Pannoramic Confocal digital slide scanner (3DHISTECH, Budapest, Hungary) in transmission mode. Images were processed with CaseViewer software (3DHISTECH). Yellow arrows showing cell infiltration along blood vessels in the lung of mice treated with UniCAR T cells and biotinylated trastuzumab. [file Image_6.jpeg]

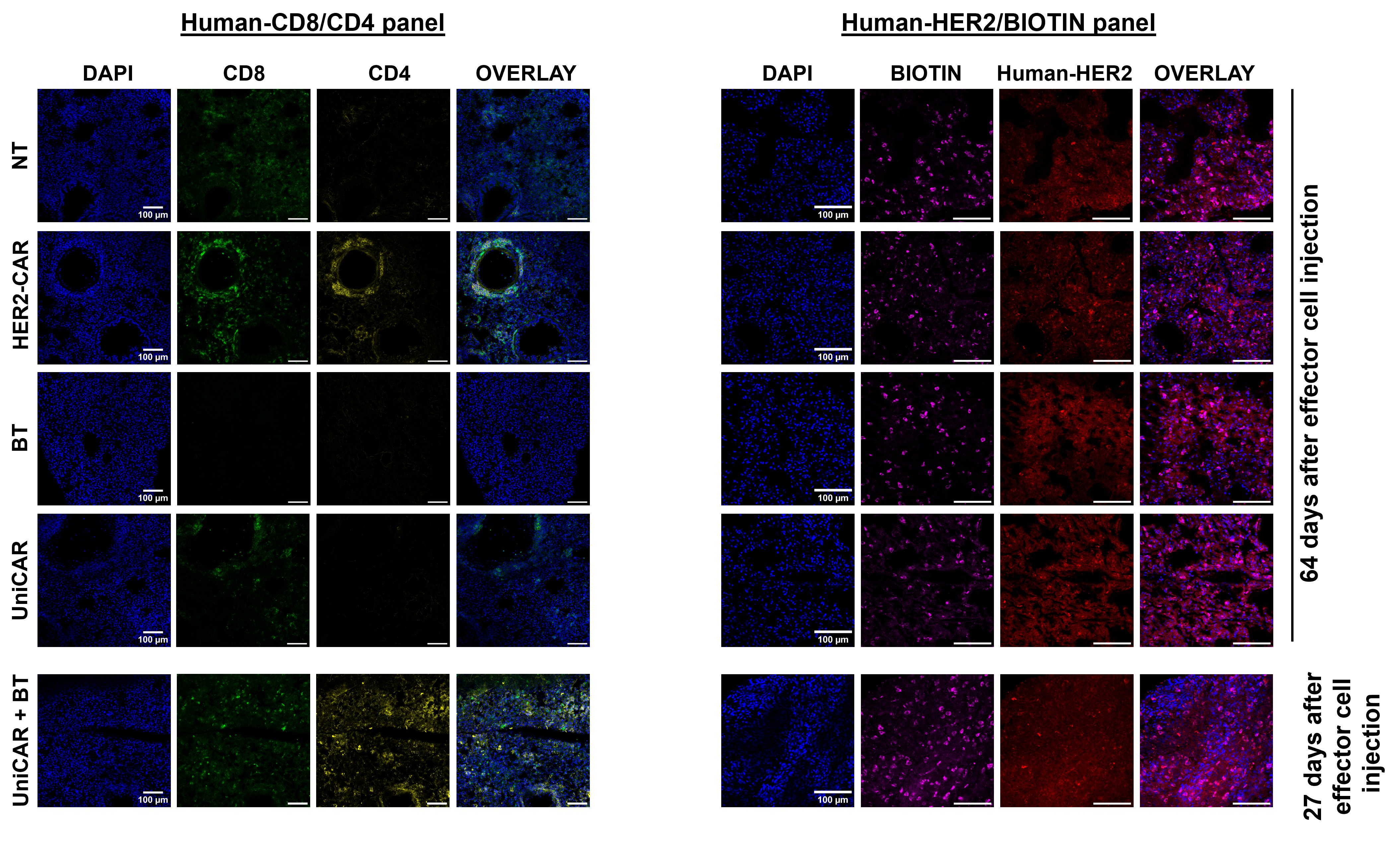

Supplement: Supplementary Figure 7 — Fluorescent images of all lung samples probed for CD8, CD4, biotin and human HER2. Representative frozen sections of mice lungs 27 days (UniCAR + BT) or 64 days (all other groups) after effector cell injection. The CD8/CD4 panel (left) shows human CD8+ cytotoxic T cells (green) and human CD4+ helper T cells (yellow) infiltrated in the lung. The biotin/human HER2 panel shows native biotin (magenta) and HER2 in the lung. Images were taken with a Zeiss LSM880 confocal fluorescence microscope. Image processing was done with Fiji ImageJ 1.53t software. [file Image_7.jpeg]

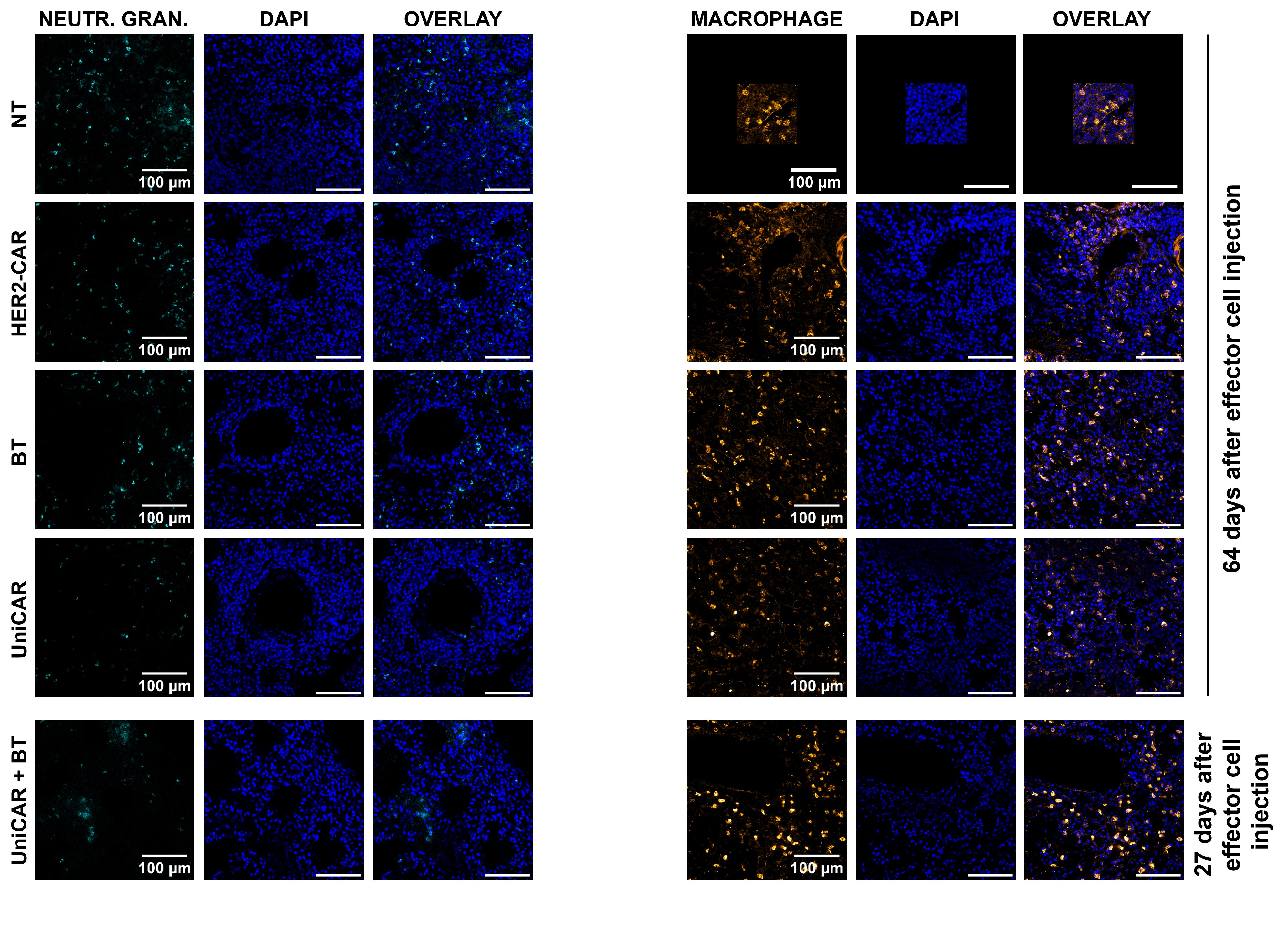

Supplement: Supplementary Figure 8 — Comparative table of all mouse neutrophile granulocyte and mouse macrophage micrographs from the lung samples. Images were taken with a Zeiss LSM880 confocal fluorescence microscope. Images show the presence of mouse neutrophil granulocytes (cyan) and mouse macrophages (orange) Image postprocessing was done with Fiji ImageJ software. (orange). Image processing was done with Fiji ImageJ 1.53t software. [file Image_8.jpeg]
